# Trends in Spirituality and Spiritual Care in Nursing—A Discursive Paper

**DOI:** 10.1111/jan.70231

**Published:** 2025-09-18

**Authors:** Fiona Timmins, Josephine Attard, Beata Dobrowolska, Michael Connolly, Sílvia Caldeira, Stelios Parissopoulos, Enrico De Luca, Jacqueline Whelan

**Affiliations:** ^1^ School of Nursing, Midwifery and Health Systems University College Dublin Dublin Ireland; ^2^ Universidade Católica Portuguesa, Faculty of Health Sciences and Nursing, Center for Interdisciplinary Research in Health Lisbon Portugal; ^3^ Department of Holistic Care and Management in Nursing, Faculty of Health Sciences Medical University of Lublin Lublin Poland; ^4^ Our Lady's Hospice & Care Services Dublin Ireland; ^5^ Faculty of Health Sciences, University of Malta Msida Malta; ^6^ Department of Nursing University of West Attica Athens Greece; ^7^ School of Nursing and Midwifery University of Birmingham Birmingham UK; ^8^ The School of Nursing and Midwifery Trinity College Dublin Ireland

**Keywords:** nursing, religion, spiritual care, spiritual needs assessment, spirituality

## Abstract

**Aim:**

This paper outlines key developments, innovations, and milestones in the field of spirituality and spiritual care in nursing.

**Design:**

A discursive paper.

**Results:**

Nursing scholars have significantly influenced the profession and contributed to the development of nursing knowledge, particularly in the field of spirituality and spiritual care. Key research has focused on nurses' perceptions and attitudes toward spirituality, clarifying foundational spiritual concepts, and establishing a framework of core spiritual care competencies for the profession.

**Conclusion:**

Despite these advancements, significant gaps remain in nurses' knowledge, understanding, and experience in providing spiritual care. The development of agreed‐upon spiritual care competencies at the European level offers important guidance for the profession, and educational initiatives are underway to support their integration. However, the field remains in an early stage of development, and further research is needed to embed spiritual care competencies into national and international nursing policy and practice. Moreover, continued research is also essential to inform and evaluate current educational programmes and nursing interventions, and to support the translation of evidence‐based knowledge into effective spiritual care delivery.

**Implications for the Profession and/or Patient Care:**

Spiritual support is proven to be an important consideration for many patients and families globally. Imbedding spiritual care education into both undergraduate and postgraduate nursing curricula is essential to prepare nurses to address the spiritual needs of patients in healthcare settings. Structured curricula that provide clear instructions on how to recognise, assess, and respond to spiritual concerns in clinical practice can enhance nurses' competence and confidence. Embedding spiritual care into education and training helps normalise spiritual care as a component of holistic nursing, supporting its inclusion in everyday care rather than treating it as an optional or marginal practice. Such educational integration has the potential to improve the consistency and quality of spiritual care across healthcare settings.

**Impact:**

Internationally there are evident gaps in the consistent provision of spiritual care to patients and their families. These are being addressed through conceptual clarity, the agreed‐upon competencies, and enhanced educational initiatives. It is essential to continue to increase awareness among the nursing profession on the necessity of addressing spiritual care needs, within the context of cultural perspectives to ensure that value is placed on the significance of these issues on a global scale.

**Patient or Public Contribution:**

There was no patient or publication contribution in this specific commentary.

## 1 Introduction

The integration of spiritual care within nursing practice can be understood as a component of person‐centred care and humanised focus on supporting patients' search for meaning and support before death, during illness, and during health crises. Furthermore, an awareness of the need to provide spiritual support to patients can promote and enhance support of dignity and compassionate approaches to care for those patients for whom faith or spirituality is important. Spirituality, though defined and interpreted differently based on individual experiences and worldviews, is a fundamental aspect of our lives that provides meaning for some around experiences such as birth, health, illness, disability, and death. Spirituality plays a crucial protective role in health and well‐being that can alleviate challenging crises in patients, clients, families and caregivers in healthcare settings (Balboni et al. 2017). Spirituality is not a static phenomenon; rather, it is a dynamic source of strength intimately connected to a person's encounters with life stressors such as altered health, illness, distress, disability, death, dying, grief and loss. Such experiences often compel individuals to shift their worldviews and perspectives on life (Carson and Koenig 2008; Puchalski et al. 2014).

While spirituality has long been an implicit component of nursing practice, a more explicit recognition and systematic integration of the spiritual dimension into nursing theories and care practices gained prominence in the late 20th century, contributing positively to individuals' health and well‐being (Gadow 1980; Bradshaw 1994; Ross 1997; Martsolf and Mickley 1998; Narayanasamy 1999; Watson 2012). McSherry (2013) pioneered a taxonomy of spirituality, outlining eight descriptors that captured theistic and postmodern views with a phenomenological focus that helped nurses toward a greater understanding of the spiritual dimension of the human experience. At its core, spirituality is about how we navigate our existence in the world, characterised by a quest for meaning and purpose in making sense of our experiences (Weathers et al. 2016). It embodies a deep desire for connection—with us, others, and the world around us—and often entails transcendence. This sense of transcendence means that spirituality is also linked to an awareness of a presence that fosters a sense of connection to something greater than us, encompassing a metaphysical realm that surpasses the physical and ephemeral aspects of existence. This realm is often described as sacred and can relate to a diverse range of entities, locations, and individuals (Pargament and Mahoney 2009). Importantly, while spirituality may encompass religious practices for some, it is entirely possible for individuals to lead a spiritual life without being religious (Weathers et al. 2016).

This universal aspect of spirituality, for example that most people search for meaning and connection in life, is vital for nurses to understand, particularly in the context of holistic patient assessments that include the spiritual dimension. Spiritual care involves not only responding to patients' fears, struggles, and worries, but also recognising and building upon their existing sources of spiritual strength, meaning and resilience (Büssing 2021). As McSherry and Ross (2013) emphasise, spiritual care is about meeting individuals at a profound level of human experience, one that includes their hopes, values, and beliefs as well as their concerns. By acknowledging both the challenges and the resources inherent in patients' spirituality, nurses can offer truly comprehensive care that supports healing and fosters well‐being. Thus, spirituality becomes not just a response to vulnerability, but a vital element of person‐centred nursing practice.

Globally, support for patients' spirituality and spiritual care provision have been recognised as essential standards for good healthcare practice, as endorsed by professional organisations. The International Council of Nurses Code of Ethics, for example, suggested that nurses must: “promote an environment in which …, religious and spiritual beliefs of the individual, families and communities are acknowledged and respected by everyone.” (ICN 2021, 7). This statement emphasises the crucial importance of respecting diverse beliefs, a fundamental principle in healthcare. However, it lacks clear guidance on providing spiritual care and does not define its scope. To create a transformative paradigm that promotes global health and humanistic values in our practice, it is essential to actively empower nurses to deliver and integrate spiritual care into nursing practice.

In the United Kingdom (UK), the Nursing and Midwifery Council (NMC) developed standards that highlight the necessity of incorporating spiritual care into nursing: They indicated that “the nurse must carry out a needs assessment and care planning according to the patient's mental, physical, cognitive, behavioural, social, and spiritual needs” (NMC standards 2018). In addition, the NMC introduced a holistic care reflective aid ‘seeing the whole person supports better care’, encouraging the nursing profession to consider diverse factors influencing a person's health, well‐being, and recovery including mental and physical health, spiritual beliefs, cognitive health, and social and personal circumstances, thus ensuring that care is person‐centred (NMC 2024).

Notably the World Health Organization (WHO), in a preamble to the constitution (WHO 1946), defined health as a state of *spiritual well‐being* among other notable aspects. However this spiritual inclusion disappeared from its original definition of health, despite ongoing discussions driven by Islamic countries and those in the Mediterranean region (Chirico 2016). However the WHO does incorporate spirituality related aspects to WHO QOL Spirituality Religiousness and Personal Beliefs Field Test Instrument (WHO 2002). Moreover it does recognise the importance of spiritual care as a core component of palliative care, to enhance the quality of life for patients who are suffering from spiritual distress (WHO 2024). More recently, there have been calls for the WHO to more fundamentally recognise the essential role of spiritual health as the fourth dimension of health and wellness (Dhar et al. 2013; Wüthrich‐Grossenbacher 2024) to ensure a comprehensive understanding of a person's health that resonates with diverse cultural and spiritual beliefs.

This discursive paper aims to explore key developments, innovations, and milestones in the integration of spirituality and spiritual care within nursing. Rather than presenting a systematic or scoping review, the discussion is grounded in a purposive selection of influential literature spanning nursing theory, education and clinical practice. The paper critically reflects on how these developments have shaped the nursing profession's engagement with spirituality and its evolving awareness in this domain. It further highlights pivotal contributions, including landmark research, policy shifts, technological advancements, and transformative changes in nursing education and practice. In addition, the paper examines and clarifies the concept of spirituality in nursing, offering a critical analysis and conceptual evaluation informed by existing frameworks (e.g., Foley and Davis 2017).

## 2 Brief Historical Background of Spiritual Care in Nursing

Before discussing the historical patterns and trends associated with the development of spirituality and spiritual care within nursing practice, it is important to consider the reasons behind the nursing profession's interest in this subject. The rise of the biomedical approach in the 18th century simplified the understanding of the body through disease, reducing it to a mere physical entity and overlooking the holistic and spiritual dimensions, thus losing sight of the person, something which the nursing profession has consistently counteracted by espousing holistic patient care (Ross 2013; McSherry et al. 2020). Furthermore, multiple government reports have identified serious healthcare failings associated with deficits in core professional values and lapses in quality care standards, prompting critical reflection on what may be missing from our practice (Clarke 2013; Ross 2013). Among these often‐overlooked elements is the spiritual dimension of care, which is deeply intertwined with values such as compassion, dignity, respect, and holistic concern for the person. Addressing spirituality in nursing therefore not only enhances patient well‐being but also helps restore the ethical and humanistic foundations of quality care. Resultantly, there is continuous and ongoing emphasis on personalised health care that pays greater attention to psychosocial needs, including spiritual care. Additionally, political, legislative, professional, and societal changes from national, European, and global perspectives have propelled the profession's interest (European Commission 2010).

The relationship between spirituality, religion, and health has deep historical roots and remains significant today. From the practices of shamans and traditional healers to the roles of chaplains in modern medical settings, spirituality is intricately woven into the healthcare system. Throughout history, various methods such as powerful incantations, mantras, prayers, repetitive rituals, physical movements, grand ceremonies, and miraculous healings have been employed to address ailments, promote well‐being, and provide meaning in the face of death. This phenomenon is observed globally, particularly in earlier times when there was no clear distinction between medicine, healthcare, and spiritual leadership in the Western world. During the Mediaeval period, for instance, the first hospitals catering to the general populace were established by religious institutions (Koenig 2012).

The proliferation of hospital‐based care in Europe during the 1800s was primarily a charitable endeavour supported by philanthropy and religious organisations. Religious observance and practice were deeply integrated into the framework of many of these institutions, with nurses supporting patients' needs and requirements together with the growth of health care chaplaincy as a professional discipline (Swift 2014). However, societal changes, such as the rise of secularism and the decline of religious orders, have made this integration less pronounced in contemporary contexts. Nevertheless, the historical legacy of delivering religious sacraments and churches presence remains significant characteristics of numerous hospitals across Europe, particularly in regions where Christian faiths have historically prevailed (Timmins et al. 2017, 2022).

The presence and accessibility of pastoral care workers and healthcare chaplains within healthcare facilities, alongside the provision of religious sacraments and ceremonies, has rendered these elements of religious care provision commonplace in such settings (Timmins et al. 2017, 2018; Fitchett and Nolan 2015). While pastoral care workers and healthcare chaplains often operate independently, they frequently collaborate as integral members of various healthcare teams (Fitchett and Nolan 2015). Their responsibilities are extensive and varied, encompassing the support of patients, families, and healthcare staff, as well as sacramental and pastoral care (Timmins et al. 2018). Additionally, they provide an informal referral service, typically initiated by nurses and other healthcare professionals (Brady et al. 2021). This indirect approach is becoming increasingly prevalent, reflecting evolving perspectives on privacy, dignity and patient care (Brady et al. 2021), despite increasing secularism in some parts of the world, and in some cases public objection to such care (Swift 2014; Medical Independent 2013).

However, regular and multi‐faith chaplaincy services are not widely available worldwide. In Greece, when chaplaincy exists, it is usually provided by clergy or trained volunteers, but formal certification and integration into clinical teams are rare. Spiritual care is typically informal, often relying on local religious leaders or the patient's own spirituality, reflecting the country's strong ties to the Greek Orthodox Church. While structured chaplaincy programmes are limited, there is growing interest in spiritual support, especially in palliative care, where collaboration with local priests is more common—though still inconsistent across facilities. These practices, rooted in Orthodox Christian traditions, contrast with the more interfaith and institutionalised models seen elsewhere and may not fully address the needs of non‐Orthodox patients (Fradelos et al. 2022).

Healthcare chaplaincy provides important and critical pastoral support that may also incorporate faith‐based elements when necessary (Kirchoff et al. 2021; O'Donovan 2011; Shields et al. 2015). However, there is growing recognition that spiritual care is a multidisciplinary responsibility (Timmins et al. 2017). While many nurses in both European and American contexts play a valuable role in making chaplaincy referrals, their involvement in spiritual care extends beyond this facilitative function. Nurses are often the first to notice spiritual distress and may engage directly in providing spiritual support through presence, active listening, compassionate communication, and attending to patients' existential concerns and emotional needs (Timmins and Caldeira 2019). These interactions can help patients find meaning, maintain hope, and feel respected and supported in their beliefs and values (Timmins et al. 2018). Despite this potential, inconsistencies persist in how nurses perceive and practise spiritual care. Some may not view it as part of their role, which can lead to limited confidence and insufficient skills in this area (Timmins and Caldeira 2019).

## 3 Transformative Developments in Nursing

Although the provision of spiritual care as a core nursing competency has not been uniformly recognised on an international scale (Pastrana et al. 2021), a growing body of evidence over the past decades has underscored the essential role nurses play in addressing the spiritual needs of patients and families. Pioneering research by Wilf McSherry and colleagues has been instrumental in mapping this evolving understanding (Cockell and McSherry 2012; McSherry and Jamieson 2011; McSherry et al. 2002; McSherry 1998, 2000, 2006). Early studies revealed widespread uncertainty and inconsistency in nurses' understanding of spirituality and spiritual care (McSherry et al. 2004; McSherry and Ross 2002; McSherry 1998), highlighting the need for clearer conceptual frameworks and education. Over time, this research contributed to a more structured discourse, informing both policy and practice and supporting the gradual integration of spirituality into nursing education and clinical competencies.

A belief in and commitment to the importance of person‐centred holistic care, particularly concerning the provision of spiritual care, does not suffice to ensure the delivery of high‐quality healthcare. Early observations by scholars indicate that while most nurses recognise the importance of spiritual care within healthcare interactions and actively provide such care, they often lack specific direction and guidance (Jones et al. 2021; Austin et al. 2016; Cockell and McSherry 2012; McSherry and Jamieson 2011). Additionally, concerns have been raised in the UK regarding the tendency of nurses to offer spiritual and/or religious care based on their personal preferences rather than those of the patient and family (Rudgard 2017). In response to these concerns, the Royal College of Nursing developed a pocket guide on Spirituality for nurses (RCN 2011), followed by an online resource for nurses providing spiritual care (Rogers and Wattis 2015).

A significant development within the nursing profession has been the establishment of clearly defined professional spiritual competencies for nurses and midwives to discern and engage in relevant interventions about spiritual care provision and alignment with patients' needs. Early contributors to the conceptual understanding of competency requirements include René Van Leewan and Joesphine Attard (Attard et al. 2014; van Leeuwen and Cusveller 2004; van Leeuwen et al. 2006, 2009).

In 2020, following 3 years of dedicated efforts within the framework of a successful Erasmus Plus project, a group of European and global experts, under the leadership of Wilf McSherry, successfully established a set of four core competencies for the first time (van Leeuwen et al. 2021). This European Erasmus Plus Project‐*Enhancing Nurses and Midwives' Competence in Providing Spiritual Care through Innovation, Education and Compassionate Care* (EPICC 2016–2019), has provided clear guidance through the development of a Spiritual Care Education Standard and core understandings of knowledge, skills and attitudes for nurses to navigate and support patients' spirituality underpinned by Intrapersonal and Interpersonal spirituality competence, Spiritual Care Assessment, Planning and Evaluation competence, and Spiritual Care Intervention and Evaluation competence.

How nurses view and think about spirituality influences their self‐awareness and shapes responses to patients and families expressing spiritual needs (Baldacchino 2015). Nurses understanding of spiritual care is essential for assessing patients spiritual care requirements and implementing appropriate spiritual care interventions (EPICC 2024; van Leeuwen et al. 2021). Within this framework (EPICC 2024) nurses are expected to identify spiritual needs and resources, plan effective interventions, evaluate health outcomes, and document the entire process (Giske et al. 2021). Nurses need to recognise their limitations concerning spiritual care interventions and to seek expert help and resources when necessary (Giske et al. 2021). The ability to assess patients' spiritual needs is fundamental to providing effective spiritual care (Karadag and Yüksel 2021). These competencies clarify nurses' roles, responsibilities, and requirements and can be used to underpin curriculum development and competency standards for nursing students to foster professional growth (van Leeuwen et al. 2021).

Projects such as EPICC provide the right direction to regions of Europe that have not yet fully addressed spiritual care competencies. For example, while spirituality is valued culturally in Greece, nursing curricula rarely cover spiritual competencies explicitly. This aligns with the broader European trend where competencies are increasingly outlined but not uniformly implemented (Rykkje et al. 2022). For example, a mixed‐method study by Fradelos et al. (2024) investigated Greek nurses' experiences with spiritual care and argued that nurses provided spiritual support primarily through existential care, emphasising empathy, respect, and holistic patient understanding rather than explicit religious care interventions. Although this study found that a nurse's educational level and the spiritual climate in healthcare settings had a positive impact on the provision of spiritual care, no reference was made to nurses' acquisition of spiritual care competencies.

Internationally, identifying and addressing spiritual care needs of patients is an essential preliminary step in the person‐centred process. Various mnemonic strategies have been proposed in the literature (Timmins and Caldeira 2017); however, evidence supporting their practical application is scarce. Consequently, a less structured approach is advocated. Prominent scholars in the field, Ross and McSherry (2018), have introduced an innovative methodology known as the 2 Question Spiritual and Holistic Assessment Model (2Q‐SAM) that proposes two questions aimed at eliciting patients' spiritual needs in care encounters: What is important to you right now? How can we help?

An understanding of how to recognise and respond to patients' spiritual needs is essential for nurses seeking to deliver holistic care. McSherry's significant contributions have provided clarity around what spiritual care entails and offer a helpful framework for its delivery in clinical settings. Addressing spiritual needs begins with the nurse's ability to assess and engage with the patient meaningfully. The following actions can support spiritual care when guided by patient needs and preferences:

- Listening actively to patients and families when spiritual concerns or questions arise
- Demonstrating sensitivity and respect for diverse beliefs and values
- Recognising spiritual distress or needs as part of a holistic nursing assessment
- Spending time with patients and families when existential or after‐life concerns are discussed
- Being attentive and present during conversations that touch on meaning, purpose, or belief
- Supporting patients and families during important spiritual seasons or rituals within their tradition
- Respectfully handling religious or spiritual items significant to the patient or family
- Providing a quiet or sacred space for reflection, prayer, or meditation
- Facilitating access to religious services or spiritual practices upon patient request
- Referring to chaplaincy or pastoral care services when additional or specialist spiritual support is needed
- Providing comfort and relief in alignment with the patient's spiritual and existential needs (Caldeira and Timmins 2017)

Concept analyses conducted by researchers like Murgia et al. (2020), Weathers et al. (2016), and Sessanna et al. (2007) have significantly contributed to knowledge and understanding of spirituality. Historically, spirituality was often seen as vague and elusive, leading to considerable confusion between spirituality and religion (Paley 2008). Achieving a clearer distinction between the two has been a crucial step in improving nurses' comprehension and practice of spirituality.

Moreover, Sílvia Caldeira has significantly deepened our awareness and understanding of spiritual distress (Caldeira et al. 2013, 2015, 2016) with a clear definition of this concept: ‘State of suffering related to the impaired ability to experience meaning in life through connectedness with self, others, world or a Superior Being’ (Caldeira et al. 2013, 82). This definition contains the attributes of spiritual distress: “suffering, impaired spirituality, contrary to spiritual well‐being, and related to meaning in life” (Caldeira et al. 2013, 82).

Enhancing nurses' understanding of spirituality is crucial for developing reliable measurements that can assess the spiritual needs of patients and their families, as well as facilitating research in this domain. The process of operationalisation—shifting from conceptual frameworks to specific inquiries in tool development—presents significant challenges. This complexity is underscored by Koenig and Carey (2024), who assert that numerous instruments are influenced by indicators of mental health, including concepts such as meaning and purpose, peacefulness, harmony, strength and comfort. As a result, research investigating the relationship between spirituality and health, particularly mental health, risks falling into tautological reasoning. Furthermore, this challenge may also emerge when spirituality is defined using terms that align more closely with the concepts of dignity, care, or compassion. By gaining a conceptual grasp of spirituality, spiritual care, and spiritual distress, nurses are empowered to practise with both confidence and competence. This empowerment is crucial in sensitive fields like spirituality, where confusion, misunderstanding, and conflict prevail.

Contemporary education of nurses has seen advancements through new thinking, to consider how students come to know spirituality with a view to responding effectively to patients and their families (Snowden and Ali 2017; Whelan 2019) and engaging in collaborative European projects. One notable initiative, the Cure to Care project (2021), focused on digital education and spiritual support within hospital settings. Developed under the Erasmus Plus programme, this project provided essential online training for nurses, particularly in the areas of palliative care and end‐of‐life care. The programme emphasised the development of two vital competencies often lacking in nursing curricula: digital competencies and religious‐spiritual competencies in a multicultural context. This latter objective is to equip nurses with the skills that address patients' religious and spiritual needs effectively, reflecting the diversity of today's society.

## 4 The Importance of Advancing Spiritual Care as A Core Nursing Competency

Lazzarino and Papadopoulos (2023) argue that the growing focus on spirituality in healthcare research and training reflects a broader shift toward the humanisation of care, emphasising a holistic, person‐centred model. Within this shift, spiritual competence becomes a key component of culturally sensitive and compassionate care (Cochrane et al. 2019), and cannot be underestimated (Connolly 2024). Families frequently report that spiritual support enables them to navigate difficult and distressing circumstances (Panicker and Ramesh 2019). As a deeply embedded cultural and human dimension (Murgia et al. 2020), spirituality, including religious or faith‐based rituals, can provide comfort, meaning, and internal strength, particularly in end‐of‐life care (Chen et al. 2018). Spiritual or religious coping mechanisms are especially vital in the context of receiving unexpected or life‐altering diagnoses and during traumatic or existential crises (Koenig 2013). In such moments, conversations around death and dying often prompt deep spiritual reflection, offering space for spiritual growth and connection for patients, their families, and healthcare providers alike (Zumstein‐Shaha et al. 2020).

Spirituality plays a crucial role in nurturing hope among patients with chronic illnesses, significantly enhancing their overall well‐being (Sabanciogullari and Yilmaz 2021). However, spiritual care should not be assumed or delivered without engaging the patient in dialogue. On the contrary, it requires sensitive assessment and respect for individual preferences. This underscores the importance of nurses' competencies in spiritual care, such as the ability to recognise, assess, and respond appropriately to patients' expressed spiritual needs, as outlined in the EPICC framework (van Leeuwen et al. 2021). Although there are cultural differences globally, families in the Republic of Ireland (ROI) for example, who have experienced death, have viewed spiritual support as particularly important (Ó Coimín et al. 2019). Moreover, one ROI Ombudsman report found the absence of care from a healthcare chaplain at the end‐of‐life was a cause of complaint (Office of the Ombudsman 2014). It is important to note that these cultural and/or religious requirements are determined from a cultural point of view. Historically, ROI populations were primarily of the Roman Catholic faith. This means that many important rituals and sacraments related to life transitions, including death, form part of the cultural heritage and tradition of the ROI.

In contrast to some countries, where spirituality and religion may be seen as separate, in other cultures with different spiritual and religious traditions, these elements are deeply intertwined with daily life and care practices. For instance, in many Asian countries influenced by Buddhism, spirituality plays a central role in palliative and end‐of‐life care. Take the People's Republic of China as an example: here, spirituality is often viewed through the lens of community‐based philosophies rather than formal religion (Zeng et al. 2023). Cultural and religious traditions have become so embedded in everyday life that it is difficult to separate religious beliefs from cultural practices. As one study explains, participants do not clearly distinguish between religion and culture. Some values, such as filial piety, are seen both as cultural traditions valued by Chinese society and as important teachings from Confucianism (Niu et al. 2021).

Spirituality in the Chinese context often includes meaning‐making, connection, transcendence, and existence. These ideas are shaped by Chinese culture and influenced by Confucian values and views on nature (Zeng et al. 2023, 3270). Unlike many Western settings, religious rituals are generally not central to spiritual support for patients and families. It remains unclear how widely spiritual care is seen as part of the nurse's role in China. Although recent studies call for nurses to help identify patients' spiritual needs (Du et al. 2022; Wang et al. 2022), research shows low levels of education and awareness in this area among nurses (Zeng et al. 2023; Li et al. 2021), as well as limited understanding of patients' spiritual needs (Wang et al. 2024).

The cultural and religious framing of spirituality by nurses in the literature is notably influenced by the geographical focus of existing studies, many of which originate from Asian and Middle Eastern contexts. In these regions, research on spirituality in healthcare has grown significantly in recent years (Heydari et al. 2016; Rafii et al. 2016). In Islamic cultural contexts, spirituality and religion are often deeply intertwined, permeating all aspects of life, including professional practice (Ahmad and Khan 2016; Heydari et al. 2016; Jafari et al. 2013). Studies involving Muslim student nurses reveal that their religious beliefs significantly influence their clinical behaviours and decision‐making (Dewi et al. 2025). In Islamic settings, the distinction between religion and spirituality is often blurred, and it has been called for the development of practical care models that better integrate spirituality into healthcare delivery (Abu‐El‐Noor and Abu‐El‐Noor 2021).

In Thailand, nurses and palliative care professionals have developed aspects of spirituality aligned with holistic nursing practices that focus on caring for the whole person (Sukcharoen et al. 2020). They emphasise the importance of integrating spirituality into the practice of self‐care and caring for others, making it a practical part of everyday life and professions. Recent ethnographic studies conducted in the Thai context have shown that spiritual care is deeply integrated into the daily routine of a palliative care centre (Davis et al. 2023). The study reported that healthcare and nursing staff believe that Buddhist principles play a significant role in providing meaning and offering solutions to moral dilemmas and stress in palliative care. The study revealed that spiritual aspects are integrated into daily practices, guiding actions, reflections, and the way patients and professional issues are handled. The entire community, including patients, family members, caregivers, and staff, actively participates in care activities right from the start. They engage in collective spiritual support through activities such as meditation, singing, several types of massage, and other shared practices (De Luca et al. 2022). These studies have also hypothesised that palliative care staff can effectively alleviate the challenges of a strict biomedical approach to spirituality by adopting a spiritual perspective on death and dying, viewing it as a natural part of life.

What is evident is that cultural and historical religious rituals are an essential element in end‐of‐life transitions for patients and their families. During this time, people seek comfort, solace, and connection through practices of imagery, poetry, music, and lighting candles, all elements commonly found in historical religious faith ceremonies. When faced with serious illness or approaching death, patients and families often seek this form of support. Although this type of support can be provided from external sources, some are preferable, but not always practical or feasible in a dynamic healthcare environment. Nurses are invariably positioned to facilitate requests for spiritual support. By nurses developing competence in this field, they are well positioned to assess patients and families' needs and provide essential support and/or referral as needed (Chirico and Nucera 2020, 2194).

## 5 Future Challenges for the Nursing Profession

In addition to more fully supporting patients and their families, the integration of spiritual care into nursing practice can support nurses' job satisfaction, as the spiritually competent nurse experiences less job‐related stress and burnout (Galea 2014; Connerton and Moe 2018; Hu et al. 2019). Nurses trained to address patients' spiritual needs and provide spiritual care positively affect patient‐reported outcomes (van de Geer et al. 2017; Karaman et al. 2022) increasing patient well‐being and satisfaction (Harorani et al. 2022). Conversely, when patients' spiritual needs are unmet, lower quality of care and decreased patient satisfaction have been reported (Best et al. 2020; Roze des Ordons et al. 2018).

Recent literature further identifies three key antecedents to developing spiritual competence in nursing: a nurse's willingness to learn about spirituality, engagement in spiritual education, and participation in spiritual‐care training. These factors are influenced by individual spiritual, religious, or philosophical beliefs and may be particularly relevant in non‐religious or secular contexts, where personal values and cultural perspectives provide the foundation for spiritual care (Alshakhshir et al. 2025). Importantly, the voluntary nature of such training is critical—mandating participation may hinder effectiveness, whereas willingness to engage is positively linked with the degree of competence attained. The outcomes of enhanced spiritual competence are both personal, such as increased well‐being, satisfaction, and personal growth, and professional, including improved patient health outcomes. These outcomes reflect spirituality's beneficial role in fostering meaning, purpose, and connection in both nurses and patients (Alshakhshir et al. 2025).

However, there is limited evidence of spiritual care education and training for nurses (McSherry et al. 2020), and limited directions in practice for assessing patients' spiritual needs and providing spiritual care. The competencies and educational resources outlined in this discursive paper constitute a starting point for improving this situation. The provision of spiritual care in the absence of education and standards is entirely problematic (Rudgard 2017). Moreover, research is required to examine the impact of spiritual care nursing interventions more closely in healthcare (Okere et al. 2024).

Fundamental questions regarding the nature of religion and spirituality and how these intersect with health and healthcare services remain. More research is needed to determine the role of faith‐based healthcare, if any, in the future of modern nursing and medicine in healthcare (Carey 2012; Pesut et al. 2012). The sustainability of healthcare chaplaincy services is uncertain (Pesut et al. 2012). Given the persistent spiritual needs of patients and families, especially at the end‐of‐life, nurses' roles are likely to expand to meet these needs, regardless of patients' religious beliefs (Du et al. 2022; Wang et al. 2022; Chirico and Nucera 2020). There is an urgent demand for specialised education for nurses (Jones et al. 2021). While digital technology can assist, sensitive end‐of‐life conversations will still require face‐to‐face support (Drummond and Carey 2020; Harrison and Scarle 2020).

One of the main challenges facing the nursing profession in the area of spirituality and spiritual care is the changing attitude toward its importance. As spiritual care becomes more integrated into nursing practice, many nurses may be expected to provide it without adequate training or preparation. Moreover, demographic changes and global health trends mean that there will be nuanced spiritual demands from an increasingly aged population living with chronic health conditions. People are known to become more spiritual as they age due to a process known as gerotranscendence (Tip et al. 2024; Atchley 2003).

Simultaneously, migration and displacement are shaping contemporary healthcare landscapes, bringing patients with diverse spiritual beliefs, values, and practices into nursing care. For many, spirituality is a vital source of strength and identity during upheaval and transition. These realities demand culturally sensitive and spiritually competent care that can support individuals navigating both existential questions and major life disruptions. Together, the intersecting trends of ageing, end‐of‐life decision‐making, and global migration call for a nursing approach that is both globally informed and individually responsive, ensuring that spiritual care is inclusive, adaptable, and integrated into everyday practice.

What is more, digital health technologies, such as telehealth, AI, and electronic health records, are reshaping how care is delivered, presenting both challenges and opportunities for spiritual care and education in nursing (Burgos et al. 2022). While digital tools can risk depersonalising care and reducing opportunities for spiritual connection, they also offer new possibilities. For example, electronic systems can prompt spiritual assessments, and AI may help identify patients in spiritual distress. Telehealth can connect patients with faith or community leaders, especially in remote areas. As digital healthcare expands, it is essential that spiritual care remains integrated, ensuring that technological advancement does not come at the cost of human connection.

To meet and adapt to these future demands, the nursing profession needs to advance its interest in the development of research and evidence‐based practice in the field of spirituality and spiritual care. Education and training of nurses are necessary, with increased use of digital technology. Although there are significant challenges ahead, this creates the opportunity to engage in strong and increasing scholarship in this area with the presence of a motivated and committed critical mass of scholars essential for future leadership in the spiritual field. There is also strong inter‐alliance between various health, faith, and non‐faith groups, curricula change and movement, and emerging support at national and international levels for the development of specific spiritual care competencies for nurses.

## 6 Conclusions and Recommendations

The need to create an integrated and supportive framework for inclusion of spiritual care in nursing and healthcare settings warrants discussion and review. Given the growing recognition of spirituality as a key dimension of healthcare (Steinhauser et al. 2017; Balboni et al. 2017), there is a critical need to establish supportive conditions and sustainable funding to advance its integration through research, education, and clinical application. Organisations and review committees must urgently recognise the importance of spirituality and spiritual care as research topics, crucial to health science, social impact, and to make a difference to people's lives.

It is also important to open an understanding of spirituality in nursing not only from palliative and cancer care perspectives but equally as a dimension of life itself, that encompasses birth, wellness, illness, to death, grief, bereavement, disability and loss. Such an approach opens up more challenges and opportunities for education, clinical care, and research, as main evidence is related to end‐of‐life care.

Transitioning from multidisciplinarity to interdisciplinary collaboration is a necessary next step. While spiritual care is a shared responsibility, it is important to acknowledge the unique expertise of spiritual care professionals such as chaplains. All healthcare workers should understand team competencies and work collaboratively to provide the most appropriate and effective support to patients. The provision of meaningful, holistic care should take precedence over any one individual delivering care in isolation. Each healthcare professional must be aware of their specific responsibilities and limitations in order to work ethically and within the scope of their competencies.

To work in healthcare and education organisations which are living laboratories where everything can change is another condition. We can only make changes we want to see if the organisational culture as a whole is sensitive to implementation. Clinical environments have the potential to promote workplace spirituality and a spiritual management culture where those who care feel cared for. In addition, the curricula must reflect the missions of the higher education institutions or nursing schools, which include educating nursing leaders to provide holistic, competent, compassionate, and holistic person‐centred care.

## 7 Implications for the Profession and/or Patient Care

- Spirituality and spiritual care are acknowledged as important aspects of nursing practice, as it relates to people's diverse cultures, health, and well‐being, giving hope, helping patients and families to cope.
- As spiritual care is a distinct aspect of holistic person‐centred care, its provision and application are fundamental to reflect what patients' and families' are seeking, supporting lives during challenging times, especially at the end‐of‐life and during bereavement.
- Effective spiritual care begins with the nurses' self‐reflection and understanding of spirituality, along with a clear grasp of the concept and its impact on care provision.
- Nurses need to be sensitive to and understand the diverse ways in which patients express their spiritual needs, and should always approach conversations or assessments with respect, openness to patients' worldviews, and their willingness to engage.
- Nurses need to understand the concept of spiritual care, to develop knowledge of the spiritual assessment process to plan care, work alongside healthcare chaplains, and know when to make timely referrals.
- Consideration needs to be directed towards improving ongoing education preparation, skills, and attitudes to effectively assess, plan, implement, evaluate, and document spiritual care provision aligned to time constraints.
- Organisations need to consider how they can best support and care for staff's spiritual care needs through mentoring.
- There is a need for healthcare systems, professionals, and educators to consider how to effectively support the spiritual needs of individuals and families who do not identify with any religious tradition, ensuring inclusive and person‐centred care for all.

These implications should be considered by nursing professionals, educators, healthcare organisations, and policy‐makers to ensure that spiritual care is embedded meaningfully into practice, education, and leadership at all levels.

## 8 Impact

- This discursive paper provides some guidance on spirituality, spiritual care, and spiritual care standards and assessment developments in nursing.
- It highlights key future challenges for the nursing profession and considers ways to address these.
- This discursive paper highlights that more research is needed to explore the impact of spiritual care in practice, in education and patients' lives.

## 9 Limitations

While this paper aims to explore key developments, innovations, and milestones in the integration of spirituality and spiritual care within nursing, several limitations must be acknowledged. First, the historical framing of nursing's relationship with spirituality is limited in scope and could benefit from deeper exploration of earlier and more diverse traditions. Second, although the paper takes a broadly international perspective, it does not fully examine existing culturally grounded models of care, such as those in Aotearoa New Zealand and the Pacific, that already integrate spirituality in meaningful ways. Additionally, the paper does not delve into complex ethical issues such as assisted dying or abortion where nurses may be directly confronted with existential questions. Finally, while we emphasise the importance of spiritual awareness in nursing, we recognise that further attention to the role of personal belief systems, self‐awareness, and the need for professional supervision would strengthen the discussion. These areas merit deeper exploration in future work.

## Ethics Statement

Ethical approval is not a requirement of commentaries.

## Conflicts of Interest

There are no conflicts of interest.

## Data Availability

Data available upon reasonable request.
